# Nicotine and cotinine enhance SARS-CoV-2 entry through distinct but complementary mechanisms in human respiratory epithelial cells

**DOI:** 10.1128/jvi.00971-26

**Published:** 2026-07-31

**Authors:** Jiheng Xu, Huei-wei Chan, Rui Yang, Xue-Ru Wu, Priyangi Malaviarachchi, He Wang, Xuming Zhang, Moon-shong Tang

**Affiliations:** 1 Division of Environmental Medicine, Department of Medicine, Pathology, and Urology, New York University Grossman School of Medicine 12296, New York, New York, USA; 2 Department of Microbiology and Immunology, University of Arkansas for Medical Sciences 318223, Little Rock, Arkansas, USA; 3 Department of Pathology, Renaissance School of Medicine, Stony Brook University 200793, Stony Brook, New York, USA; Loyola University Chicago - Health Sciences Campus, Maywood, Illinois, USA

**Keywords:** nicotine, cotinine, SARS-CoV-2, viral-cell-entry, viral-spike-protein cleavage

## Abstract

Previous epidemiological studies have shown that E-cigarette and tobacco users are more likely to develop COVID-19 symptoms than non-users. To investigate the underlying mechanisms, we examined the effects of nicotine, the major component of tobacco and E-cigarette, and its major metabolite, cotinine, on the susceptibility of human respiratory epithelial cells to SARS-CoV-2 infection. We found that pre-treatment with nicotine and cotinine significantly and additively enhanced viral infection. While nicotine increased the expression of the viral receptor ACE2 and the serine protease TMPRSS2, cotinine upregulated cysteine protease cathepsin B and promoted viral spike protein cleavage. These findings suggest that nicotine and cotinine enhance SARS-CoV-2 infection at the cell entry stage through distinct mechanisms. Using a SARS-CoV-2 pseudovirus system, we further investigated the effects and mechanisms of nicotine and cotinine on viral entry. We found that both compounds enhanced pseudovirus infection, but with different time courses. Nicotine’s effect correlated with the upregulation of ACE2 and TMPRSS2, whereas cotinine’s effect corresponded with increased cathepsin B and viral spike protein cleavage. The cathepsin B inhibitor E64d completely abolished cotinine-enhanced viral spike protein cleavage and viral entry. In contrast, ACE2 and TMPRSS2 inhibitors (chloromethylketone and camostat) had limited effects on viral spike protein cleavage and only partially reduced the viral entry enhancement induced by nicotine and cotinine. These results indicate that nicotine promotes virus-receptor binding and cell entry, while cotinine facilitates viral entry through cathepsin B-mediated spike protein cleavage.

**IMPORTANCE:**

This study highlights the potential risks of tobacco and E-cigarette use in increasing susceptibility to COVID-19. We show that the major neurostimulant in tobacco and E-cigarette, nicotine, and its metabolite, cotinine, additively enhance SARS-CoV-2 infection in human respiratory epithelial cells by promoting viral entry. Specifically, nicotine and cotinine upregulate the expression of distinct, yet complementary sets of key cellular proteins required for viral entry. These findings suggest that both tobacco smoking and E-cigarette vaping may exacerbate COVID-19 infection rates and severity and provide new insights into how nicotine and cotinine contribute to viral susceptibility. This research underscores the need for public health measures to address the heightened risk posed by tobacco smoking and E-cigarette vaping to encounter the SARS-CoV-2 infection.

## INTRODUCTION

The global SARS-CoV-2 pandemic has caused significant disruption to daily life, economic activities, and social interactions. Fortunately, the rapid development of effective vaccines and treatments has greatly reduced the health risks associated with this virus. The emergence of more infectious but less virulent SARS-CoV-2 Omicron subvariants and more recently the NB.1.8.1 strain has further contributed to reducing the severity of infections (1–3). However, SARS-CoV-2 infection can still lead to severe outcomes, including death, particularly in older adults, individuals with underlying health conditions, and tobacco smokers (4–12). Numerous epidemiological studies have shown that smokers who contract COVID-19 experience more severe symptoms and higher mortality rates compared to nonsmokers (5–14). Intriguingly, results of several studies from China suggest that tobacco smokers are underrepresented among hospitalized SARS-CoV-2 patients (8, 10, 11, 15), sparking speculation about a protective or therapeutic role for nicotine, a major component of tobacco smoke (15–17). Epidemiological studies on electronic cigarette (E-cig) use and COVID-19 have also yielded conflicting results. While some studies report no significant difference in the prevalence or severity of COVID-19 symptoms between E-cig users and non-users (18, 19), others suggest that E-cig users, particularly young adults, are more likely to experience more severe COVID-19 symptoms (7, 13). Currently, there are an estimated 1.25 billion tobacco smokers and 82 million E-cig users worldwide (20, 21). If tobacco smoke and E-cig vaping impact susceptibility to SARS-CoV-2 infection—whether positively or negatively—this constitutes a significant public health concern that warrants thorough investigation for risk assessment and designing preventive strategies. The inconsistent results from epidemiological studies underscore the urgent need for experimental investigations to clarify the effects of tobacco smoke and E-cig vaping on SARS-CoV-2 infection and to uncover the underlying biological mechanisms.

The primary purpose of smoking tobacco and using E-cigs is to inhale nicotine, a neurostimulant constituted in both tobacco smoke and E-cig aerosols. In conventional tobacco smoking, nicotine (10–12 mg/cigarette) is released by burning the tobacco leaves, while in most commercially available E-cigs, nicotine (15–36 mg/mL) is aerosolized from a solution of propylene glycol (PG) and vegetable glycerin (VG) through controlled heating (22, 23). Tobacco smoke contains not only nicotine but also numerous byproducts of incomplete combustion. In contrast, E-cig aerosols consist primarily of nicotine, PG, and VG, with few, if any, tobacco-specific components (22–24). Once inhaled, approximately 80% of nicotine is rapidly metabolized into cotinine *in vivo*. Nicotine has a half-life of 2–4 h, while cotinine has a longer half-life of 16–24 h (25).

To explore how tobacco smoking and E-cig vaping affect COVID-19 susceptibility, we first assessed the effects of nicotine and cotinine on SARS-CoV-2 infection in the immortalized human bronchial epithelial cell (HBEC) line BEAS-2B, primary cultured HBECs, and physiologically relevant three-dimensional air-liquid interface human airway epithelial (ALI-HAE) cultures, which are considered primary targets of initial viral infection. We then examined how nicotine and cotinine influence the expression of the viral receptor angiotensin-converting enzyme 2 (ACE2), which is essential for viral binding, and transmembrane serine proteinase 2 (TMPRSS2), which cleaves the viral spike protein (VSP) to facilitate fusion between viral envelope and host cell membrane during entry (26–30). Additionally, we investigated the effects of nicotine and cotinine on the expression of endosomal-lysosomal cysteine protease cathepsin B (CTSB) and its role in VSP cleavage, a critical step for viral-cell membrane fusion. Finally, we investigated the potential relationship between protein expression, VSP cleavage, and viral entry by employing specific inhibitors and a SARS-CoV-2 pseudovirus model. Our results demonstrate that nicotine and cotinine indeed promote SARS-CoV-2 infection at the initial stage of the viral life cycle, that is, cell entry, through upregulation of distinct yet complementary sets of cellular proteins.

## RESULTS

### Nicotine and cotinine treatments enhance the susceptibility of human bronchial epithelial cells to SARS-CoV-2 infection

**Fig 1:**
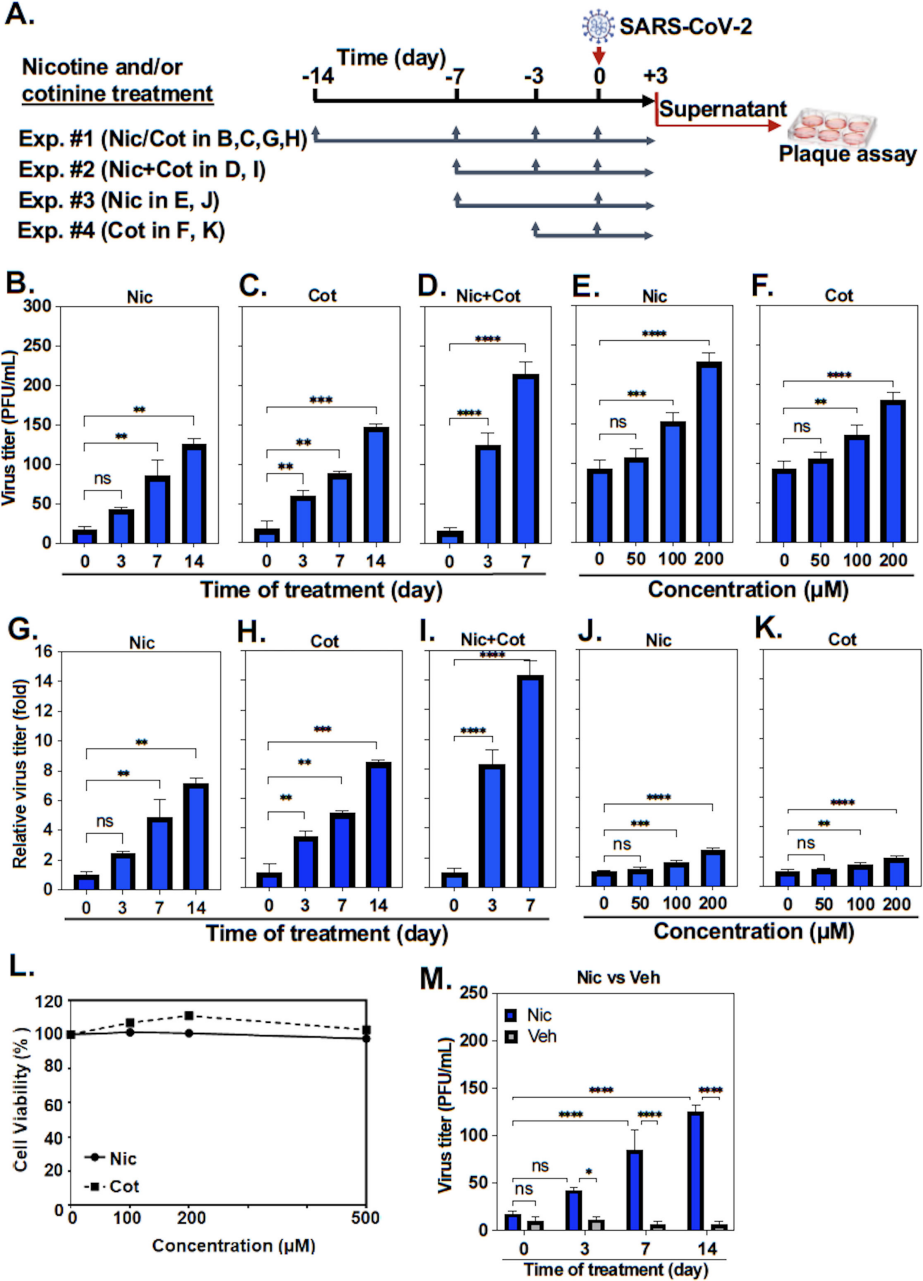
Nicotine and cotinine enhance BEAS-2B cell susceptibility to SARS-CoV-2 infection. (**A**) Experimental flowchart. Immortalized BEAS-2B cells were treated with (1) 100 µM of nicotine (Nic) or cotinine (Cot) for 3, 7, and 14 days (**B, C, G, H**); (2) both nicotine and cotinine at 100 µM each for 3 and 7 days (**D, I**); (3) 50 to 200 µM nicotine for 7 days (**E, J**); or (4) 50 to 200 µM cotinine for 3 days (**F, K**). After treatments, cells were infected with SARS-CoV-2 at a multiplicity of infection (MOI) of 1 for 1 h. Infected cells were incubated with nicotine and/or cotinine at the same concentrations for 3 days. Virus titers in the culture medium were quantified by plaque assay and expressed as PFU/mL (**B–F**) or relative titer (fold change) (**G–K**) compared to untreated controls (0 day or 0 concentration). (**L**) Cytotoxicity of nicotine and cotinine in BEAS-2B cells. Exponentially growing cells were treated with various concentrations of nicotine (Nic) or cotinine (Cot) for 24 h at 37°C. Cell survival was assessed using the WST-1 assay and expressed as percentage (%) relative to the untreated control, which is set as 100%. (**M**) To determine the effect of E-cig vehicles (PG and VG) on viral infectivity, BEAS-2B cells were treated with: (i) 100 μM PG and 100 μM VG (Veh) or (ii) 100 μM of nicotine (Nic) for 0, 3, 7, and 14 days. Following treatments, cells were infected with SARS-CoV-2, and the virus titers were determined. Data are representative of two to three independent experiments. Bars represent standard deviation. ns: not significant; *, **, ***, and **** represent *P*-value < 0.05, 0.01, 0.001, and 0.0001, respectively, as determined by one-way ANOVA (**B–K**) or two-way ANOVA (**M**).

Since the respiratory tract is the primary site of both SARS-CoV-2 infection and nicotine absorption from inhaled tobacco smoke and E-cig aerosols, we used immortalized cells BEAS-2B as surrogates to investigate the effects of nicotine and cotinine on cell susceptibility to SARS-CoV-2 infection. Immortalized BEAS-2B cells were treated with 100 µM nicotine and/or cotinine for up to 14 days and then infected with SARS-CoV-2, as described in Materials and Methods and shown in Fig. 1A. The results demonstrated that pre-treatment with nicotine and cotinine significantly enhanced SARS-CoV-2 titer in a time-dependent manner, resulting in a two- to eightfold increase in infectious virus production from days 3 to 14 (Fig. 1B and G; Fig. 1C and H). Combined treatment with nicotine and cotinine resulted in an additive effect, increasing infectious virus production by 8- and 14-fold on days 3 and 7, respectively (Fig. 1D and I). Both nicotine and cotinine enhanced infectious virus production in a concentration-dependent manner (Fig. 1E and J; Fig. 1F and K). Importantly, this concentration range of nicotine and cotinine did not affect cell viability (Fig. 1L).

Since in commercial E-cig, nicotine is dissolved in PG and VG solvents and aerosolized with controlled heat to generate “misty” vapors, it is possible that PG and VG vehicles may affect SARS-CoV-2 infection. To test this possibility, BEAS-2B cells were treated with PG and VG for durations (0, 3, 7, and 14 days) the same as for nicotine and cotinine treatments. As shown in Fig. 1M, neither PG nor VG treatment had any significant effect on viral infection. Together, these findings indicate that nicotine and cotinine, but not the E-cig solvents PG and VG, may promote viral infection in immortalized BEAS-2B through different mechanisms.

**Fig 2:**
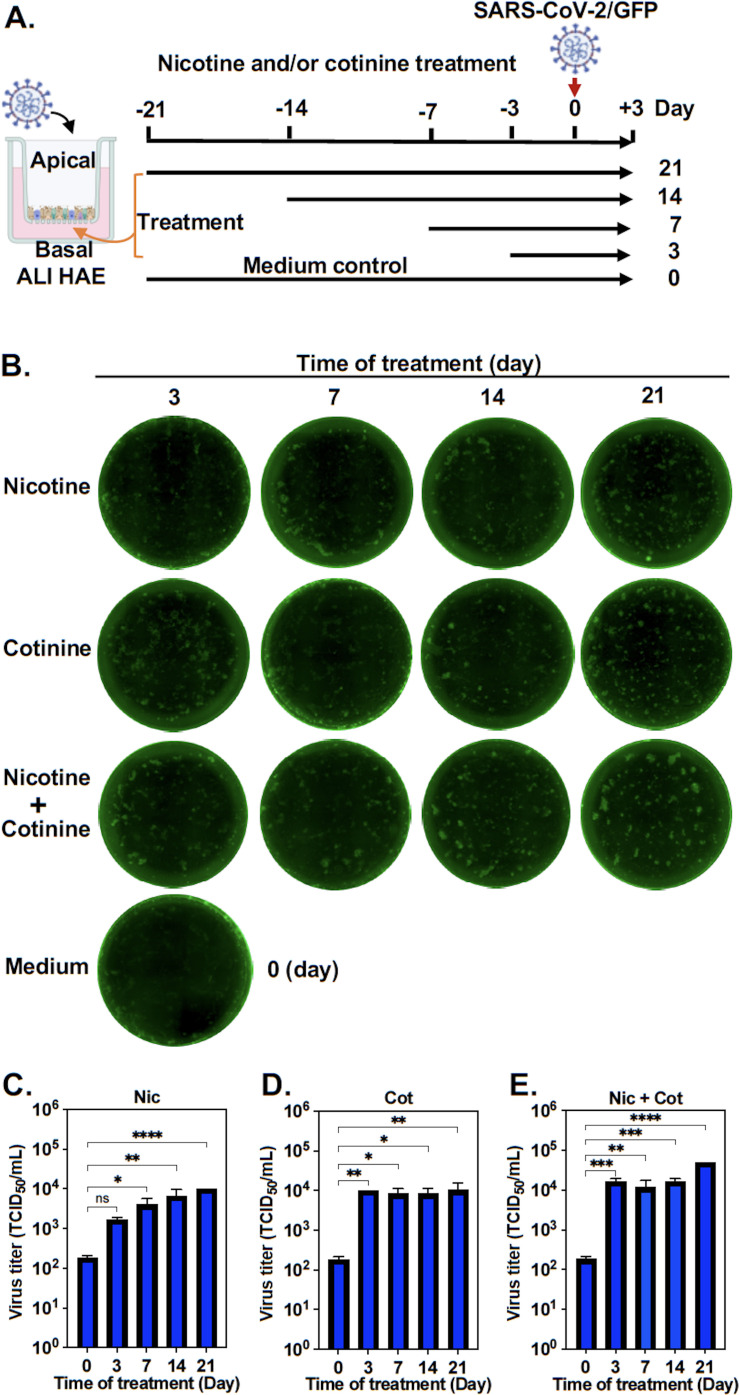
Nicotine and cotinine enhance the susceptibility of differentiated air-liquid interface human airway epithelial (ALI-HAE) cultures to SARS-CoV-2 infection. (**A**) Schematic illustration of the air-liquid interface culture system and the experimental timeline for nicotine/cotinine treatment and SARS-CoV-2/GFP infection. ALI-HAE cultures were treated with nicotine, cotinine, or a combination of both compounds at a concentration of 100 µM each for 3, 7, 14, or 21 days prior to viral infection. Untreated ALI-HAE cultures served as controls. (**B**) Effect of nicotine/cotinine treatment on GFP expression. Representative fluorescent images of ALI-HAE cultures showing the extent and intensity of GFP expression at 3 days post-infection following treatment with nicotine, cotinine, or both compounds, compared with untreated controls. (**C–E**) Effect of nicotine and/or cotinine treatment on infectious virus production. Shed virus was collected from the apical surface of ALI-HAE cultures at 3 days post-infection following treatment with nicotine, cotinine, or both compounds, and virus titers were determined by TCID_50_ assay and expressed as TCID_50_/mL. The 0-day treatment group represents untreated controls. Error bars represent the standard deviation (SD) of the mean (*n* = 3). ns: not significant; *, **, ***, and **** indicate *P*-value < 0.05, 0.01, 0.001, and 0.0001, respectively, as determined by one-way ANOVA.

To further determine whether the effects of nicotine and cotinine observed in immortalized BEAS-2B cells are also relevant in human airway epithelium, we established three-dimensional ALI-HAE cultures (Fig. 2A). These cultures were differentiated from primary human bronchial/tracheal epithelial cells (HBTECs) and recapitulate the multicellular architecture and cellular composition of the human airway epithelium *in vivo* (31, 32). ALI-HAE cultures were treated with nicotine, cotinine, or a combination of both compounds in the basolateral chamber at a concentration of 100 µM each for 3, 7, 14, or 21 days prior to SARS-CoV-2 infection and maintained throughout the 3-day post-infection period. The cultures were then infected apically with SARS-CoV-2/green fluorescent protein (GFP) (33), a recombinant virus expressing green fluorescent protein (GFP) (Fig. 2A). As shown in Fig. 2B, nicotine- and/or cotinine-treated cultures generally exhibited more intense and widespread GFP-positive foci than untreated controls at 3 days post-infection. Although the size, number, and intensity of fluorescent foci varied considerably among treatment groups and between individual inserts, the enhancement of infection was generally more pronounced following 21 days of treatment than shorter exposure periods (Fig. 2B). To quantify infectious virus production, apical washes were collected at 3 days post-infection, and viral titers were determined by the standard 50% tissue culture infectious dose (TCID_50_) assay. Nicotine treatment significantly increased viral titers following 7–21 days of exposure (*P* < 0.05), with the magnitude of enhancement increasing with treatment duration, whereas no significant effect was observed after 3 days of treatment (*P* > 0.05) (Fig. 2C). In contrast, cotinine treatment significantly enhanced viral titers after only 3 days of exposure (*P* < 0.05), and viral titers remained at similar elevated levels following up to 21 days of treatment (Fig. 2D). Moreover, cultures treated with both nicotine and cotinine generally produced higher viral titers than those treated with either compound alone (Fig. 2E). Collectively, these findings demonstrate that both nicotine and cotinine enhance SARS-CoV-2 infection in immortalized BEAS-2B cells and in physiologically more relevant primary ALI-HAE cultures.

### Nicotine and cotinine have differential effects on the expression of ACE2, TMPRSS2, furin, and CTSB

Because viral entry depends on binding of the viral spike protein (VSP) to ACE2 (34), the primary receptor for SARS-CoV-2, and because previous studies have demonstrated increased ACE2 expression in the airways of current tobacco smokers and individuals with chronic obstructive pulmonary disease (35), as well as in small animal models exposed to tobacco smoke or E-cig vapor (36–38), we hypothesized that nicotine and cotinine may enhance SARS-CoV-2 infection by modulating ACE2 expression. In addition to ACE2, host cell proteases such as TMPRSS2, furin, and cathepsins B/L (CTSB/L) are critical for VSP activation and subsequent viral entry (26–30). We therefore investigated the effects of nicotine and cotinine on the expression of these key proteins.

**Fig 3:**
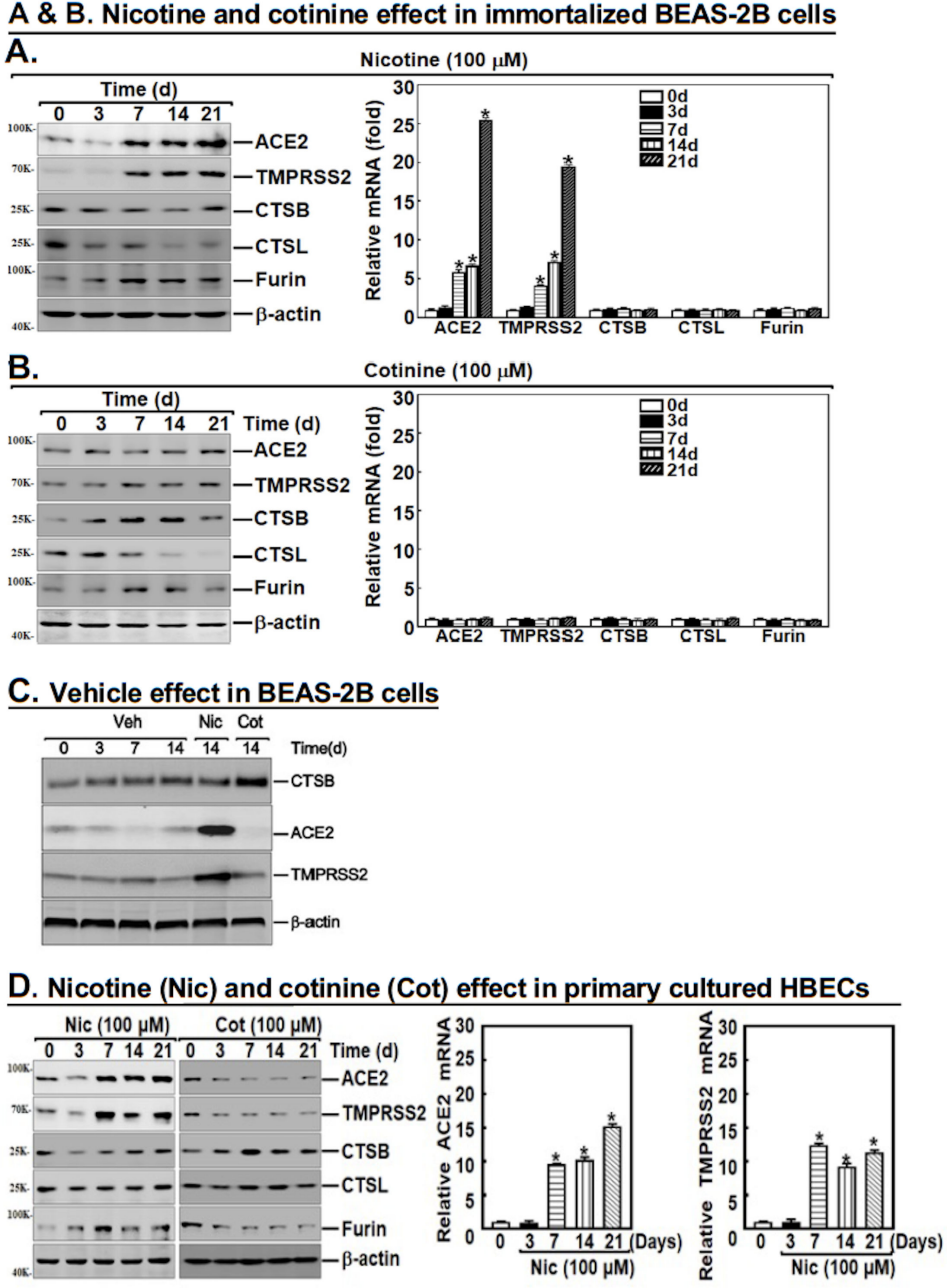
Effects of nicotine, cotinine, and E-cig vehicle (PG and VG) on the expression of ACE2, TMPRSS2, furin, CTSB, and CTSL in immortalized BEAS-2B cells, and primary HBECs. BEAS-2B or primary HBECs were treated with 100 μM nicotine (Nic) or cotinine (Cot) for 3 to 21 days (**A, B, and D**) or E-cig vehicles (PG and VG, 50% each) for 3–14 days (**C**). 0 day represents untreated control. Expression of ACE2, TMPRSS2, furin, CTSB, and CTSL was assessed at the protein level by Western blot and at the mRNA level by quantitative real-time-PCR. Bars represent standard deviations. * represents *P*-values < 0.05.

Our results show that nicotine treatment (i) upregulates ACE2 and TMPRSS2 at both protein and messenger RNA (mRNA) levels in a time-dependent manner; (ii) increases furin protein expression in a time-dependent manner, without affecting its mRNA levels; and (iii) downregulates cathepsin L (CTSL) expression, with no effect on CTSB expression (Fig. 3A). In contrast, cotinine treatment had no effect on ACE2 or TMPRSS2 expression but significantly upregulated CTSB and furin at the protein level in a time-dependent manner, without changes in mRNA levels (Fig. 3B). The results in Fig. 3C show that vehicle (PG + VG) treatment does not have any significant effect on the expression of ACE2, TMPRSS2, and CTSB. These results are consistent with the findings shown in Fig. 1M that vehicle treatment alone does not enhance SARS-CoV-2 infection.

Because immortalized BEAS-2B cells are transformed with SV40 and harbor sequestered p53 protein due to the SV40 T antigen, the immortalization process could potentially influence cellular responses to nicotine and cotinine and affect viral susceptibility. To test this possibility, we also evaluated the effects of nicotine and cotinine in primary cultured HBECs. Consistent with results in immortalized BEAS-2B cells, nicotine treatment in primary HBECs increased the expression of ACE2, TMPRSS2, and furin in a time-dependent manner, while cotinine specifically upregulated CTSB expression but had no effect on ACE2 or TMPRSS2. Unlike immortalized BEAS-2B cells, cotinine treatment in primary HBECs downregulated furin expression. Neither nicotine nor cotinine affected CTSL expression in primary HBECs (Fig. 3D). Overall, these findings indicate that nicotine enhances viral entry by transcriptionally upregulating ACE2 and TMPRSS2, promoting virus-receptor binding and entry via the plasma membrane. In contrast, cotinine post-transcriptionally upregulates CTSB (and furin in immortalized BEAS-2B), promoting viral fusion with the endosomal membrane. Both pathways are critical for efficient SARS-CoV-2 cell entry (26–30).

### Nicotine and cotinine enhance SARS-CoV-2 pseudovirus entry via different time courses

**Fig 4:**
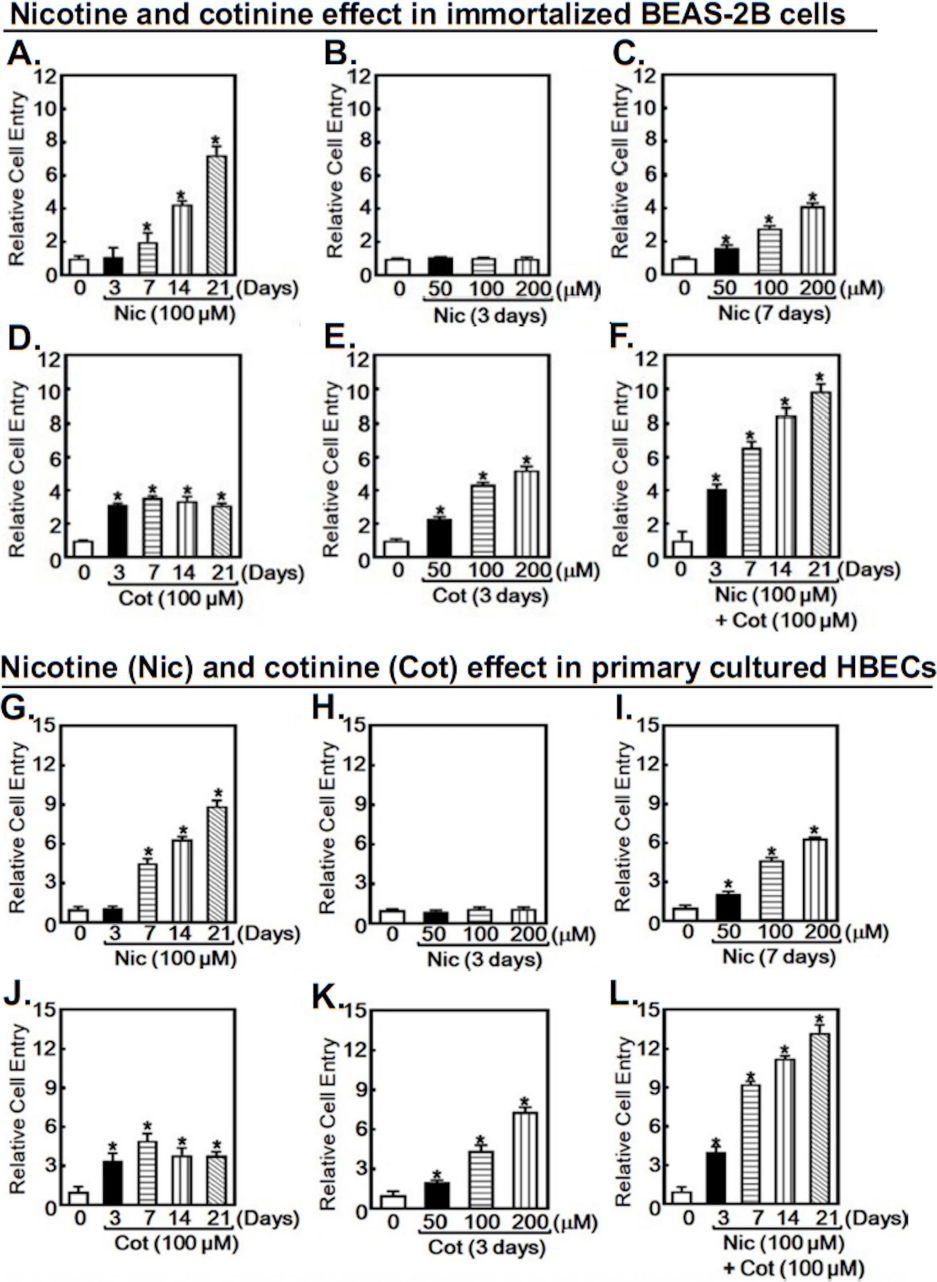
Nicotine and cotinine enhance SARS-CoV-2 pseudovirus cell entry. Immortalized BEAS-2B cells (upper panels [**A–F**]) or primary HBECs (lower panels [**G–J**]) were treated with nicotine (Nic) (**A–C, G and H**), cotinine (Cot) (**D and E**; **J and K**), or both (**F and L**) at 100 µM each for 3 to 21 days, as indicated, or with different concentrations of nicotine for 3 days (**B and H**), 7 days (**C and F**), or cotinine for 3 days (**E and K**). Cells were then infected with an equal amount of SARS-CoV-2 pseudovirus in the presence of nicotine and/or cotinine for 48 h. Luciferase activity was measured in cell lysates, and pseudovirus entry efficiency was expressed as relative luciferase activity compared to untreated controls. Bars represent standard deviation. * represents *P*-values < 0.05.

To determine whether the increased infectious virus production observed with nicotine and cotinine treatments (Fig. 1 and 2) resulted from enhanced viral entry through upregulation of cellular receptors and proteases (Fig. 3), we employed the SARS-CoV-2 pseudovirus model. This pseudovirus shares the same viral envelope and VSP as the authentic SARS-CoV-2 but contains a luciferase-expressing retrovirus vector instead of the SARS-CoV-2 genome, allowing quantitative assessment of viral entry through luciferase gene expression (27). BEAS-2B cells were pre-treated with nicotine and cotinine (100 μM each) for 0 to 21 days, followed by infection with an equal amount of SARS-CoV-2 pseudovirus. The efficiency of viral-cell entry was then assessed by measuring luciferase activity in the infected cells. As shown in Fig. 4A, nicotine treatment did not affect pseudovirus entry after 3 days but significantly increased entry after 7 to 21 days, aligning the time course of nicotine-induced ACE2 and TMPRSS2 expression (compare Fig. 4A to Fig. 3A). Furthermore, nicotine increased pseudovirus entry in a dose-dependent manner after 7 days of treatment, but not after 3 days (Fig. 4B and C).

In contrast, cotinine treatment enhanced pseudovirus entry much earlier. A three- to fivefold increase was observed as early as 3 days post-treatment (Fig. 4D), and this effect was dose-dependent (Fig. 4E), consistent with the cotinine-induced upregulation of CTSB (Fig. 3B). Notably, while CTSB expression continued to rise through day 7, the enhancement in pseudovirus entry remained stable from day 3 to day 21 (Fig. 3B and 4D). This suggests that viral load may become a limiting factor during pseudovirus infection.

When nicotine and cotinine were combined, pseudovirus entry increased additively: an 8-fold increase at day 3 and a 14-fold increase at day 7 (Fig. 4F), approximately matching the sum of the individual effects of nicotine and cotinine observed at corresponding time points (compare to Fig. 4A and D).

We also evaluated the effects of nicotine and cotinine on pseudovirus entry in primary cultured HBECs. The results mirrored those observed in immortalized BEAS-2B cells, with both nicotine and cotinine enhancing pseudovirus entry in a dose- and time-dependent manner (compare the upper panels (A–F) with the lower panels (G–L) of Fig. 4). Taken together, these findings demonstrate that nicotine and cotinine promote the initial stage of the SARS-CoV-2 life cycle, that is, cell entry, through distinct but complementary mechanisms.

### Cotinine enhances VSP cleavage and pseudovirus entry

**Fig 5:**
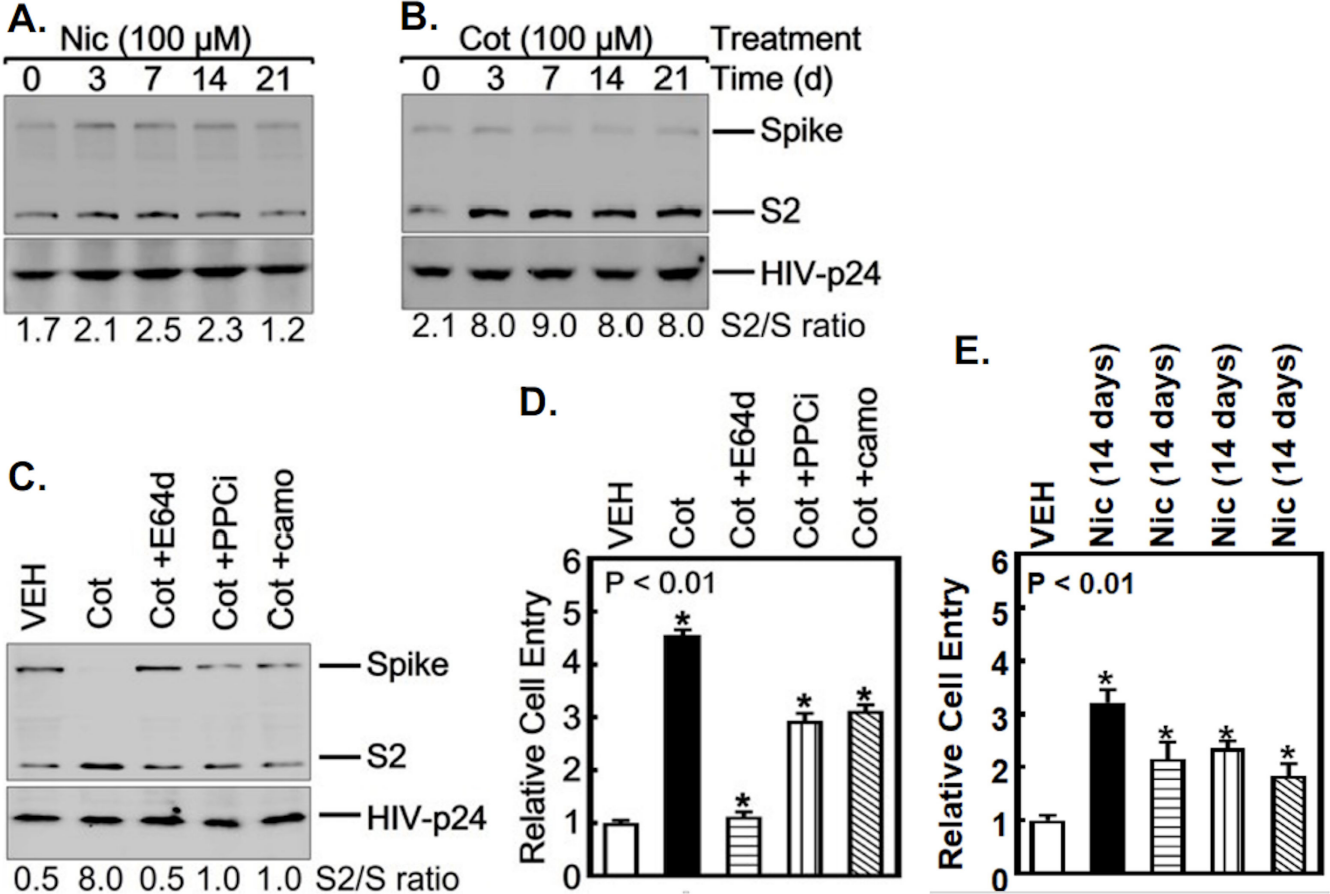
Effect of nicotine, cotinine, and protease inhibitors on SARS-CoV-2 spike protein (VSP) cleavage and pseudovirus cell entry. HEK293T cells were treated with nicotine (Nic) (**A**) or cotinine (Cot) (**B**) at 100 μM for 3 to 21 days, followed by transfection with plasmids to generate SARS-CoV-2 pseudovirus for 48 h. Packaged pseudoviruses were analyzed for viral spike protein (VSP) cleavage by Western blot. In panels C, D, and E, HEK293T cells were pre-treated with cotinine (Cot) at 100 μM for 3 days (**C** and **D**), or nicotine (Nic) at 100 μM for 14 days (**E**), in the absence and presence of the CTSB inhibitor E64d (50 µM), the TMPRSS2 inhibitor chloromethylketone (PPCi, 5 µM), or the furin inhibitor camostat (camo) (50 µM), followed by transfection with the equal amount of the same plasmids as in panels A and B. Pseudoviruses were analyzed for VSP cleavage (**C**) and relative cell entry efficiency in immortalized BEAS-2B cells by luciferase assay (**D** and **E**). Cell entry efficiency was quantified by measuring relative luciferase activity compared to the vehicle (VEH) control. HIV-p24 was used as a loading control. Full-length spike protein (S) and cleaved S2 subunit (S2) are indicated. S2/S ratios were determined by densitometry. Bars represent standard deviations. * represents *P*-values < 0.05.

We next investigated the effects of nicotine and cotinine on the activation of VSP. SARS-CoV-2 pseudovirus was generated in HEK293 cells pre-treated with nicotine or cotinine as described in Materials and Methods. The cleavage status of the VSP was then assessed. As shown in Fig. 5A and B, nicotine treatment slightly enhanced VSP cleavage, increasing the subunit S2 to full-length S (S2/S) ratio from 1.7 to 2.5. In contrast, cotinine treatment significantly enhanced VSP cleavage, as indicated by a higher S2/S ratio (from 2.1 to 9). Notably, this enhancement of VSP cleavage followed a time course parallel to the elevation of CTSB and the enhancement of pseudovirus entry observed with cotinine treatment (Fig. 5B vs Fig. 3B and 4D).

We then used inhibitors of CTSB, furin, and TMPRSS2 to determine the roles of these specific proteases induced by nicotine and cotinine in VSP cleavage and viral-cell entry enhancement. HEK293T cells were treated with 100 μM cotinine or nicotine for 3 days and then transfected with plasmids to generate SARS-CoV-2 pseudovirus. The transfected cells were incubated for an additional 2 days to allow pseudovirus maturation in the presence of the CTSB inhibitor E64d, the furin inhibitor chloromethylketone (PPCi), or the TMPRSS2 inhibitor camostat (camo). The resulting pseudovirus was used to assess VSP cleavage (Fig. 5C) and infectivity in immortalized BEAS-2B cells (Fig. 5D). The results show that E64d treatment completely blocked cotinine-enhanced VSP cleavage (S2/S ratio reduced from 8 to 0.5) and also abolished cotinine-enhanced pseudovirus entry. In contrast, TMPRSS2 and furin inhibitors partially blocked cotinine-enhanced VSP cleavage (S2/S ratio reduced from 8 to 1) and pseudovirus entry (≈35%). These results confirm that cotinine enhances viral-cell entry by promoting CTSB-mediated VSP cleavage, facilitating viral fusion with the endosomal membrane. Additionally, these protease inhibitors also partially inhibited nicotine-enhanced viral cell entry (35%–50%) (Fig. 5E). Taken together, these results (Fig. 1 to 5) demonstrate that both nicotine and cotinine enhance SARS-CoV-2 infection at the viral-cell entry step. Nicotine primarily enhances entry through the upregulation of ACE2 and TMPRSS2, which facilitates virus-receptor binding and subsequent entry, while cotinine enhances entry by promoting CTSB-mediated spike protein cleavage and fusion between the viral envelope and the endosomal membrane.

## DISCUSSION

We have shown that nicotine and cotinine treatments significantly enhance the susceptibility of immortalized BEAS-2B cells, primary cultured HBECs, and ALI-HAE cultures to SARS-CoV-2 infection in a time- and concentration-dependent manner (Fig. 1 and 2). Additionally, we demonstrated that nicotine and cotinine increase the expression of key proteins—ACE2, TMPRSS2, and CTSB—in BEAS-2B and primary cultured HBECs (Fig. 3). Since ACE2, TMPRSS2, and CTSB are essential for virus-receptor binding, VSP cleavage, and virus-cell membrane fusion—all critical steps for viral entry (27–29, 39)—our findings suggest that the enhanced viral infectivity observed is likely due to increased cell entry facilitated by the upregulation of these cellular receptors and proteases. Consistent with this interpretation, we observed that nicotine and cotinine treatments significantly increased the susceptibility of BEAS-2B and primary cultured HBECs to SARS-CoV-2 pseudovirus entry (Fig. 4). Thus, our data establish that tobacco smoke and E-cig aerosols, via nicotine and its metabolite cotinine, may enhance the susceptibility of respiratory epithelial cells to SARS-CoV-2 infection by promoting viral entry through upregulation of ACE2, TMPRSS2, and CTSB.

The additive effect of nicotine and cotinine on enhancing SARS-CoV-2 infectivity (Fig. 1E and J), also confirmed in the pseudovirus system (Fig. 4F and L), suggests that their actions may be complementary rather than identical. This is further supported by our findings that nicotine and cotinine induce distinct sets of cellular proteins (Fig. 3). While nicotine treatment increases the expression of ACE2 and TMPRSS2, cotinine does not affect these proteins but instead upregulates CTSB. Furthermore, the time courses of nicotine-induced increase in ACE2 and TMPRSS2 expression correlate with enhanced pseudovirus entry (Fig. 3 and 4). Nicotine treatment only minimally affects VSP S1/S2 cleavage, and treatment with a TMPRSS2 inhibitor partially reduces nicotine-enhanced pseudovirus entry (Fig. 5). These results suggest that nicotine enhances SARS-CoV-2 infection primarily through increased virus-ACE2 receptor binding at the initial step of viral entry.

In contrast, cotinine treatment shows a time-dependent elevation of CTSB expression, which correlates with increased susceptibility to SARS-CoV-2 pseudovirus entry and enhanced viral infection (Fig. 1 to 4). Furthermore, the CTSB inhibitor E64d completely blocked cotinine-enhanced viral entry and VSP cleavage (Fig. 5), indicating that cotinine enhances SARS-CoV-2 infection by promoting CTSB-mediated VSP cleavage and viral-cell membrane fusion, the second step after receptor binding and internalization. Notably, unlike the ancestral USA-WA1/2020 isolate used in this study, the more transmissible Omicron variants preferentially utilize the endocytic route for cellular entry (40–42). Previous studies have shown that Omicron variants do not form syncytia in tissue culture—a process dependent on VSP cleavage at the plasma membrane by proteases such as TMPRSS2—and that VSP cleavage in Omicron is largely independent of TMPRSS2 (40–42). These observations suggest that cotinine may exert a greater effect on virus entry in Omicron variants than in the ancestral strain examined here. However, further studies are required to determine whether cotinine-induced upregulation of cathepsin B specifically enhances the entry of Omicron-lineage SARS-CoV-2 variants. Collectively, these findings highlight that nicotine and cotinine enhance SARS-CoV-2 infection in respiratory epithelial cells through complementary mechanisms.

We also found that both nicotine and cotinine treatments upregulate furin expression in BEAS-2B cells (Fig. 3A and B). However, in primary cultured HBECs, nicotine increases furin expression, while cotinine downregulates it (Fig. 3D). Despite this difference, both nicotine and cotinine treatments enhance SARS-CoV-2 pseudovirus entry in primary cultured HBECs (Fig. 4G through L). Furin inhibition by chloromethylketone (PPCi) partially reduced cotinine-enhanced VSP S1/S2 cleavage as well as cotinine- and nicotine-enhanced pseudovirus entry in BEAS-2B cells (Fig. 5C and E). These results suggest that while furin plays a role in VSP cleavage and viral entry, CTSB is the primary mediator of cotinine-enhanced infection.

**Fig 6:**
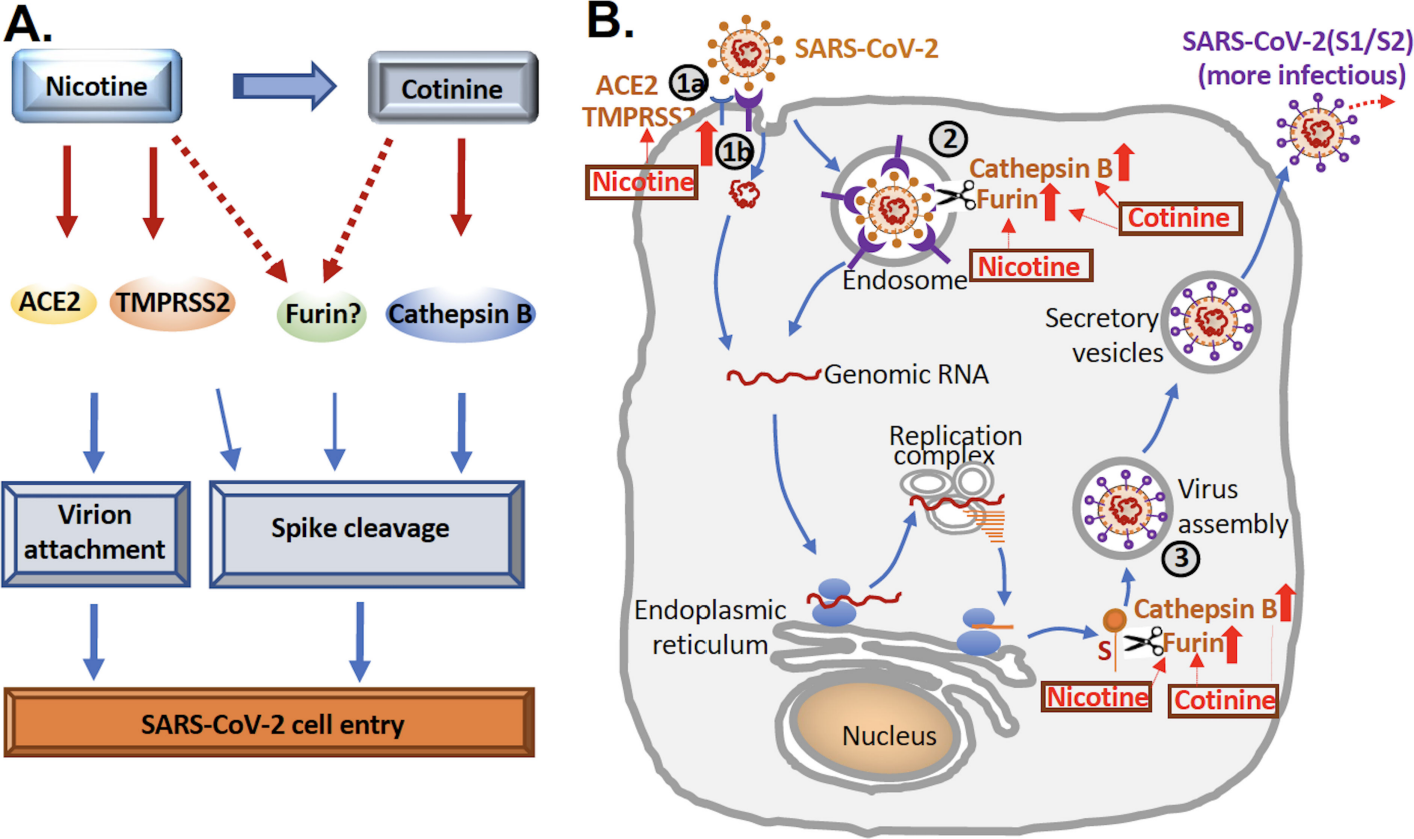
Proposed model for the effects of nicotine and cotinine on SARS-CoV-2 infection in respiratory epithelial cells. (**A**) Schematic presentation of how nicotine and cotinine influence SARS-CoV-2 cell entry through distinct and overlapping mechanisms. (**B**) Nicotine and cotinine promote SARS-CoV-2 infection at three stages of the virus life cycle: (1a) upregulation of ACE2 and TMPRSS2 by nicotine enhances SARS-CoV-2 binding and cell entry. (1b) TMPRSS2 facilitates cleavage of the spike protein and fusion of the viral envelope with the plasma membrane. (2) Surface-bound SARS-CoV-2 with uncleaved VSP is internalized via receptor-mediated endocytosis. Cotinine upregulates cathepsin B, and both nicotine and cotinine upregulate furin, increasing cleavage of the spike protein, facilitating fusion between the viral envelope and the endosomal membrane, and release of viral genome into the cytoplasm. (3) Elevated furin and cathepsin B expression by nicotine and cotinine may enhance cleavage of the spike protein, generating more infectious viruses upon subsequent infection of new target cells.

The emerging model from this study is that nicotine and cotinine promote SARS-CoV-2 infection via two distinct yet complementary mechanisms (Fig. 6). Nicotine primarily upregulates ACE2 and TMPRSS2 at the transcriptional level, while cotinine upregulates CTSB at the post-transcriptional level. Both nicotine and cotinine also contribute to the post-transcriptional regulation of furin (Fig. 3 and 6A). Increased ACE2 expression facilitates SARS-CoV-2 binding to the cell surface and enhances viral entry (step 1a in Fig. 6B). When TMPRSS2 is present or upregulated, it cleaves the VSP, enabling fusion of the viral envelope with the plasma membrane and subsequent release of the viral genome into the cytoplasm (step 1b in Fig. 6B). In the absence or at low levels of TMPRSS2, virus particles bound to the cell surface are internalized via receptor-mediated endocytosis (step 2 in Fig. 6B). Our finding that cotinine upregulates CTSB and enhances pseudovirus entry at an early time point—after 3 days of treatment, when TMPRSS2 has not yet been upregulated by nicotine—supports this alternative entry route (Fig. 3 and 6). Once inside the endosome, the elevated CTSB and, to a lesser extent, furin accelerate VSP cleavage into S1 and S2 subunits. This cleavage induces conformational changes in the spike protein that facilitate fusion with the endosomal membrane, thereby releasing the viral RNA genome into the cytoplasm for reproduction (step 2 in Fig. 6B). Additionally, increased expression of CTSB and furin during virus assembly and egress (Fig. 3 and step 3 in Fig. 6B) may enhance the infectivity and transmissibility of progeny virions, as has been reported for furin in the context of SARS-CoV-2 pseudovirus production (27). However, whether nicotine and cotinine contribute to this pathway during viral assembly and egress remains to be determined.

The mechanism by which nicotine induces TMPRSS2 and ACE2 is not fully understood. One possibility is that nicotine induces demethylation of CpG islands in the promoters of these genes, since both genes contain CpG islands that can be downregulated via enhancing activity of DNA methyltransferases (43). Another potential mechanism is the upregulation of ACE2 via the upregulation of the nicotinic acetylcholine receptor α7 (nAChR-α7) (44). Additionally, nicotine has been shown to increase androgen expression (45, 46), which in turn upregulates both TMPRSS2 and ACE2 (45). These findings suggest that nicotine may enhance SARS-CoV-2 infection more quickly and efficiently *in vivo* than observed in cultured cells.

It is well established that, in humans, 80% of nicotine is rapidly converted to cotinine and that cotinine has a much longer half-life of 16–24 h (25). Consequently, both nicotine and cotinine circulate in the blood of habitual E-cig and tobacco users at any given time. This explains why the additive effects of nicotine and cotinine observed in this study could significantly enhance viral infection. Thus, our findings support epidemiological data suggesting that tobacco and E-cig users are more susceptible to SARS-CoV-2 infection and may develop more severe COVID-19 symptoms. Moreover, we propose that SARS-CoV-2 released from infected tobacco and E-cig users may become more infectious upon transmission, potentially contributing to higher transmissibility (7, 13). The higher susceptibility of E-cig users to COVID-19 compared to tobacco smokers remains unclear and intriguing. Our results show that the vehicle for E-cig does not enhance SARS-CoV-2 infection (Fig. 1M) or the induction of viral receptors like ACE2 and the VSP cleavage proteases like TMPRSS2 and CTSB (Fig. 3C). If nicotine uptake is similar between E-cig users and tobacco smokers, the increased susceptibility of E-cig users could be attributed to other chemicals in tobacco smoke that may interfere with the function of ACE2, TMPRSS2, and CTSB. Furthermore, it has been reported that hamsters exposed to E-cig vapor exhibit upregulated ACE2 expression, along with increased levels of proinflammatory cytokines and chemokines in the lungs (38). Thus, future studies could utilize this animal model to determine whether nicotine- and cotinine-induced increases in receptor expression or proinflammatory cytokine production contribute to disease severity following SARS-CoV-2 infection.

In conclusion, our findings provide experimental and mechanistic evidence supporting epidemiological data suggesting that tobacco smoking and E-cig vaping increase susceptibility to COVID-19 and contribute to more severe disease outcomes (6, 17).

## MATERIALS AND METHODS

### Cells, virus, and reagents

Immortalized human bronchial epithelial cell line BEAS-2B (ATCC CRL-3588) and African green monkey kidney cell line Vero-E6 (ATCC CRL-1586) were obtained from American Type Culture Collection (Manassas, VA, USA). Primary cultured normal human bronchial epithelial cells (HBECs) and their specialized culture medium were obtained from Lonza Bioscience (Morrisville, NC, USA) and were used for monolayer cell culture. Dulbecco’s Modified Eagle Medium (DMEM) and fetal bovine serum (FBS) were purchased from Corning Life Sciences (Teterboro, NJ, USA). HEK239T cells were obtained from Thermo Fisher Scientific (Holtsville, NY, USA). Primary human bronchial/tracheal epithelial cells (HBTEC) (FC-0035), the BronchiaLife B/T medium complete kit (LL-0023), and the HBTEC air-liquid interface differentiation medium (LM-0050) were purchased from Lifeline Cell Technology (Frederick, MD, USA) and were used for differentiation of air-liquid interface human airway epithelial (ALI-HAE) culture. The three plasmids—SARS-CoV-2 spike, pCMV-dR8.2 dvpr, and pHIV-Luc-ZsGreen—were purchased from Addgene (Watertown, MA, USA). Antibodies against ACE2, TMPRSS2, furin, cathepsin B (CTSB), cathepsin L (CTSL), and SARS-CoV-2 spike protein S2 were purchased from Santa Cruz Biotechnology (Dallas, TX, USA), Invitrogen, and Thermo Fisher Scientific (Holtsville, NY, USA). Camostat and E64d were purchased from Sigma-Aldrich, and chloromethylketone was purchased from Enzo Life Sciences (Farmingdale, NY, USA). Nicotine, cotinine, propylene glycol, and vegetable glycerin were purchased from Sigma-Aldrich (St. Louis, MO).

SARS-CoV-2 isolate USA-WA1/2020 (NR-52281) was obtained from BEI Resources, National Institute of Allergy and Infectious Diseases, National Institutes of Health, and a recombinant SARS-CoV-2 expressing the green fluorescence protein (SARS-CoV-2/GFP) was kindly provided by Dr. Ralph Baric (UNC, Chapel Hill) (33). The viruses were propagated in Vero-E6 cells. Virus stocks were prepared from cultured supernatants at 3 days post-infection, and the virus titer in the supernatants was determined by a standard plaque assay in Vero-E6 cells (47), with the results expressed as plaque-forming units (PFU) per mL.

### Treatment of cells with nicotine, cotinine, or protein convertase inhibitors

We assume that E-cig users consuming one JUUL pod (0.7 mL, nicotine 59 mg/mL) per day would consume 41 mg of nicotine—their nicotine concentration would be less than 42 μM, with cotinine levels likely being under 34 µM, assuming a blood volume of 6 liters. In heavy tobacco smokers, consuming 20 cigarettes (a pack)/day, nicotine levels would be less than 203 µM and cotinine under 162 µM, assuming each cigarette contains 10 mg of nicotine. Therefore, we tested a range of nicotine and cotinine concentrations from 50 to 200 µM in cell cultures.

For nicotine and cotinine treatments, BEAS-2B cells and primary HBECs growing at 70%–80% confluency were treated with varying concentrations of nicotine or cotinine (dissolved in growth medium) for varying time periods (0 to 21 days) (41). Treated cells were passaged at a 1:3 ratio every 3–4 days and further cultured in the same growth medium with nicotine and/or cotinine at the same concentrations to prevent overgrowth.

For protein convertase inhibitor treatment, BEAS-2B cells or primary HBECs were pre-treated with chloromethylketone (5 μM), camostat (50 μM), E64d (50 μM), or vehicle (control) for 6 h, followed by nicotine and cotinine treatment.

### Cell viability assay

Cell suspensions were seeded into 96-well plates (100 µL/well) and treated with varying concentrations of nicotine or cotinine. The cell viabilities were detected by WST-1 assay as previously described (41).

### SARS-CoV-2 infection in BEAS-2B cells

Following treatment with nicotine and/or cotinine as indicated, BEAS-2B cells were infected with SARS-CoV-2 at a multiplicity of infection (MOI) of 1. One-hour post-infection, the virus inoculum was removed, and the cells were rinsed once with phosphate-buffered saline (PBS) before being replenished with fresh DMEM containing 2% FBS. Infected cells were then incubated for 3 days in the presence or absence of nicotine and/or cotinine at the same concentrations used during pre-treatment. Supernatants were collected to determine virus titer by plaque assay (47). Virus titers were expressed as PFU/mL and also converted to a fold change relative to the untreated or vehicle-treated control, which was set to 1.

### Air-liquid interface culture of primary human airway epithelial cells, nicotine/cotinine treatment, and SARS-CoV-2/GFP infection

Primary HBTECs were expanded and passaged twice in BronchiaLife Complete Medium. At passage 3, cells were seeded onto 24-well permeable inserts (6 mm in diameter) (Corning Costar PET Transwell, Cat. No. 3470) at a density of 5 × 10^4^ cells per insert in the apical chamber and differentiated at an air-liquid interface (ALI) using Air-Liquid Interface Differentiation Medium (Lifeline Cell Technology, Cat. No. LL-0023) according to the manufacturer’s instructions.

During differentiation, cells were treated with nicotine, cotinine, or a combination of both compounds at a final concentration of 100 μM each. Treatments were administered via the basolateral chamber by replacing the differentiation medium every 2–3 days for 3, 7, 14, or 21 days.

For viral infection, differentiated ALI-human airway epithelial (ALI-HAE) cultures were washed apically with calcium- and magnesium-free PBS. Cells were then infected from the apical surface with 100 µL SARS-CoV-2/GFP (4 × 10^4^ PFU per insert) in PBS for 2 h at 37°C. Following infection, the viral inoculum was removed, and the apical surface was rinsed with calcium- and magnesium-free PBS. Fresh basolateral medium containing nicotine, cotinine, or both compounds at the same concentrations used during differentiation was added, and cultures were maintained for an additional 3 days. The experiment was repeated three times.

At 3 days post-infection, GFP expression was examined by fluorescence microscopy. GFP-positive cells across the entire insert were imaged using a digital fluorescence microscope (BZ-X810, Keyence). To collect shed virus, the apical surface of each culture was incubated with 200 μL of calcium- and magnesium-free PBS at 37°C for 30 min. The apical washes were collected and stored at −80°C until analysis. Viral titers were determined by the standard 50% tissue culture infectious dose (TCID_50_) assay using Vero-E6 cells (48). Results were expressed as TCID_50_/mL.

### Western blot analysis

Cell lysates were prepared as described previously (49). Cellular protein expression was detected by Western blot using the specific antibodies indicated, following the protocol outlined in reference (49). To assess the VSP cleavage, both the full-length spike (S) and the cleaved S2 subunit (S2) were detected in transfected HEK293T cells using an antibody specific to the S2 subunit (27). The amounts of S and S2 were quantified by densitometry.

### Reverse transcription PCR and quantitative real-time PCR

Total RNA was extracted using TRIzol reagent (Invitrogen, Waltham, MA, USA), and complementary DNAs (cDNAs) were synthesized with the SuperScript IV First-Strand Synthesis System for RT-PCR (Invitrogen). Oligonucleotides (Forward: 5′-CAG GTG GTC TCC TCT GAC TT-3′ and Reverse: 5′-CCA AAT TCG TTG TCA TAC CA-3′) were used to amplify human GAPDH cDNA as a loading control. The ACE2 cDNA fragments were amplified with a pair of ACE2-specific PCR primers (Forward: 5′-ACA GTC CAC ACT TGC CCA AAT-3′ and Reverse: 5′-TGA GAG CAC TGA AGA CCC ATT-3′). TMPRSS2 cDNA was amplified using the primers (Forward: 5′-GTC CCC ACT GTC TAC GAG GT-3′; Reverse: 5′-CAG ACG ACG GGG TTG GAA G-3′). Cathepsin B cDNA was amplified using primers (Forward: 5′-ACA ACG TGG ACA TGA GCT ACT-3′; Reverse: 5′-TCG GTA AAC ATA ACT CTC TGG GG-3′). Furin cDNA was amplified using primers (Forward: 5′-TCG GGG ACT ATT ACC ACT TCT G-3′; Reverse: 5′-CCA GCC ACT GTA CTT GAG GC-3′). Cycle threshold (Ct) values were determined, and the expression level of each mRNA was calculated using the 2^−△△Ct^ method, expressed relative to GAPDH.

### SARS-CoV-2 pseudovirus preparation and infection

SARS-CoV-2 pseudoviruses were generated using the method developed by Shang et al. (27). Briefly, the SARS-CoV-2 pseudoviruses were generated by cotransfection of (i) the SARS-CoV-2 spike plasmid, (ii) packaging plasmid pCMV-dR8.2 dvpr, and (iii) transducing plasmid pHIV-Luc-ZsGreen into HEK293T cells at 75% confluency using PolyJetTM *In Vitro* DNA Transfection Reagent (SignaGen Laboratories, Rockville, MD, USA). Seventy-two hours post-transfection, supernatants containing mature SARS-CoV-2 pseudoviruses were collected, filtered, and frozen at −80°C. The pseudovirus was used for infection of immortalized BEAS-2B cells and primary HBECs for 48 h, and the efficiency of pseudovirus entry was determined by measuring the luciferase activity.

### Assay for luciferase activity

Following pseudovirus infection, cell lysates were prepared, and luciferase activity in the lysates was determined as described by Shang et al. (27). The relative efficiency of viral-cell entry in treated cells versus control cells was calculated by assigning the luciferase activity in control cells as 1.

### Statistical analysis

Data were analyzed using Student’s *t*-test and one-way or two-way analysis of variance (ANOVA), as appropriate.

## Data Availability

All data are included in the article.
